# Neuroinflammatory Approach to Surgical Trauma: Biomarkers and Mechanisms of Immune and Neuroendocrine Responses

**DOI:** 10.3390/jpm14080829

**Published:** 2024-08-05

**Authors:** Gustavo N. Silva, Virna G. A. Brandão, Marcelo V. Perez, Kenneth Blum, Kai-Uwe Lewandrowski, Rossano K. A. Fiorelli

**Affiliations:** 1 Department of Anesthesiology, Gaffrée e Guinle Universitary Hospital (EBSERH), Federal University of the State of Rio de Janeiro (UNIRIO), Rio de Janeiro 22290-240, RJ, Brazil; virna.brandao@unirio.br; 2 Department of Surgery and Anesthesia, Federal University of São Paulo (UNIFESP), São Paulo 04021-001, SP, Brazil; marcelo.perez@fcmsantacasasp.edu.br; 3 Division of Addiction Research & Education, Center for Sports, Exercise & Mental Health, Western University of Health Sciences, Pomona, CA 91766, USA; drd2gene@gmail.com; 4 Center for Advanced Spine Care of Southern Arizona and Surgical Institute of Tucson, Tucson, AZ 85712, USA; business@tucsonspine.com; 5 Department of General and Specialized Surgery, Gaffrée e Guinle Universitary Hospital (EBSERH), Federal University of the State of Rio de Janeiro (UNIRIO), Rio de Janeiro 22290-240, RJ, Brazil; rossano.fiorelli@unirio.br

**Keywords:** surgical trauma, biomarkers, systemic inflammation

## Abstract

The severity and invasiveness of clinical outcomes from organic responses to trauma are influenced by individual, surgical, and anesthetic factors. A stress response elicits neuroendocrine and immune reactions that may lead to multi-organ dysfunction. The degree of neuroinflammatory reflex activation from trauma can increase pro-inflammatory cytokine production, leading to endothelial dysfunction, glycocalyx damage, neutrophil activation, and multisystem tissue destruction. A shift in patient treatment towards a neuroinflammatory perspective has prompted a new evaluation protocol for surgical patients, required to understand surgical pathogenesis and its link to chosen anesthetic–surgical methods. The goal of this study is to summarize and disseminate the present knowledge about the mechanisms involved in immune and neuroendocrine responses, focusing on video laparoscopic surgeries. This article outlines various measures cited in the literature aimed at reducing the burden of surgical trauma. It reviews anesthetic drugs, anesthetic techniques, and intensive care procedures that are known to have immunomodulatory effects. The results show a preference for more sensitive inflammatory mediators to tissue trauma serving as care tools, indicators for prognosis, and therapeutic outcomes.

## 1. Introduction

It is increasingly recognized that anesthetic management can indirectly influence immunostimulatory and immunosuppressive mechanisms by modulating immune cell function or directly by mitigating the stress response through intravenous agents or regional anesthesia. The choice of anesthetic technique affects the balance between pro/anti-inflammatory responses, potentially altering clinical outcomes [1,2,3].

The implementation of minimally invasive laparoscopic surgery along with an advanced recovery protocol that utilizes multimodal analgesia has led to improved patient outcomes. These include a decrease in opioid consumption, expedited mobilization, and a faster return of bowel function [4].

Surgical tissue injury triggers a range of crucial reactions for restoring the organism’s homeostasis. An appropriate inflammatory response is vital for tissue repair. The stress response includes elevated hormone levels (ACTH, cortisol, and catecholamines), complement system activation, leukocyte migration to the injury site, and cytokine release (Interleukins and Tumor Necrosis Factor), along with other cellular products (superoxide radicals, proteases, and growth factors). This stress response can alter a patient’s immunological status [5].

Tissue damage is detected by peripheral and central nociceptors, which trigger an electrical response through the depolarization of the axonal membrane and stimulate the activation and interaction of the hypothalamic–pituitary sympathetic, endocrine, and immune systems. The electrical impulse produced by these nociceptors’ activation is conveyed via nerve fibers to the spinal cord’s dorsal horn, prompting the activation of the central nervous system and an immediate sympathetic neurological reaction [6].

IL-6, the most representative cytokine in an injury setting, serves as an indicator of the inflammatory response following surgical trauma. It triggers the liver to produce acute-phase reactants, such as C-reactive protein; stimulates neutrophil production in the bone marrow; and aids in the differentiation of T helper cells that produce IL-17. It is produced by macrophages, dendritic cells, endothelial cells, fibroblasts, and other cells in response to pathogen-associated molecular patterns, as well as IL-1 and TNF. The serum levels of these substances rise proportionally to the stress level, as seen in sepsis, leading to mitochondrial dysfunction, glycocalyx disruption, and endothelial dysfunction, all of which are associated with increased morbidity and mortality. CRP levels usually start to increase about four to six hours after surgical injury, peaking within 48 h. After uncomplicated surgeries, CRP levels generally decrease, returning to normal within 72 to 168 h. Consequently, both IL-6 and CRP levels in plasma are indicative of the extent of surgical trauma, reflecting their relationship and plasma kinetics [7,8,9].

The surgical stress response is mediated by other cytokines, catecholamines, glucagon, insulin, growth hormone, aldosterone, and antidiuretic hormone. Under stress conditions like trauma, sepsis, and cancer, an excess of pro-inflammatory cytokines disrupts the homeostasis, leading to increased morbidity and mortality [10]. Controlling stress-induced activation of the sympathetic nervous system is crucial to prevent hemodynamic compromise, hyperinflammation, coagulopathy, immune dysfunction, hypermetabolism or hypercatabolism, and hypothermia [5].

Identifying injury-sensitive markers in laparoscopic procedures could greatly advance the field. This narrative review assesses the inflammatory response and underscores the significance of certain markers employed in clinical–surgical practice, which impact clinical outcomes.

## 2. Methods

This study is a narrative review about the systemic stress response triggered by surgical trauma, like sterile inflammation preceding immune and neuroendocrine dysregulation. To this end, the authors followed search strategies and three databases widely used in the healthcare area were selected: PubMed, MEDLINE, and EMBASE. The research was conducted from February 2024 to July 2024, based on health sciences descriptors (DeCSs)—“Surgical stress and anesthesia” AND “Neuroendocrine response” AND “Tissue damage”—in their various combinations. At a certain point, the descriptors were crossed with each other. In the process, some filters were added as inclusion criteria: studies dating from 2014 to 2024, free full texts, clinical trials, systematic reviews, and observational studies. Then, 624 articles were selected for abstract reading, of which 26 corresponded to the research purpose. In addition, 19 additional articles were included in the study through a manual search.

For didactic purposes, this article is divided into systemic inflammation and tissue damage, modulation of the inflammatory response to surgical trauma, ischemic and pharmacologic preconditioning, and endothelial glycocalyx protection.

## 3. Results

### 3.1. Systemic Inflammation and Tissue Damage

Regardless of the surgical access route, the direct and indirect effects of surgery result in cell damage. Direct surgical injury occurs due to surgical manipulation, tissue mobilization, excision, and dissection, which can lead to the release of high levels of inflammatory mediators and cytokines that drive immunological, metabolic, and hormonal processes, known as the surgical stress response. Indirect injury may be caused by blood loss, changes in perfusion, microvascular alterations, and anesthetic techniques, which can affect hemoglobin concentration, cardiac output, and arterial oxygen saturation. This may result in a decrease in the supply of oxygen to the tissues, predisposing them to the development of organ dysfunction and SIRS [11].

Surgical stress is defined as an acute response to the impairment of body barrier functions due to sterile injury (incision, excision, manipulation, and pain), pathogen invasion (intestinal bacterial translocation or postoperative wound infection), and/or anesthesia. In trauma, the initial response is an increase in sympathetic discharge. The activation of the sympathetic nervous system results in the transmission of information to the cerebral cortex and thalamus through myelinated Aδ fibers and unmyelinated C fibers. If this is not contained, it has multiple effects on homeostasis, including the stimulation of inflammation, alterations in coagulation, modifications to immune competence, and T cell mobilization. This process is mediated by β2-adrenergic receptors [12].

Modulating the inflammatory response to surgical trauma via multimodal anesthesia equips anesthesiologists with potent therapeutic strategies, leading to improved postoperative clinical outcomes.

#### 3.1.1. Immune Response

Evolutionarily conserved biochemical mechanisms play a key role during exposure to surgical stress, leading to the engagement of signaling pathways in the innate and cell-mediated adaptive immune systems. The impact of surgery on the immune and neuroendocrine–metabolic systems is influenced by several factors, including the intensity of surgical trauma, malnutrition, infection, and cancer [13].

Initially, there is a complex integration between cytokines, alarmins, and physiological signals, generating the recruitment of inflammatory cells, particularly macrophages, neutrophils, and dendritic cells; the activation of Th1, Th2, and Th17 effector CD4+ helper T cells of innate immunity; and the release of natural killer (NK) cells, which are analogous to CD8+ cytotoxic T cells. These cells phagocytize the offending agent and produce additional doses of cytokines that lead to positive feedback for the activation of specific transcription factors for innate immunity cells and a possible adaptive immune response. Norepinephrine (NE) is released by the SNS and signals via β1- and β2-adrenergic receptors. The cytotoxicity of NK cells and the expression of IFNγ, granzyme B, and perforin are reduced when the signaling of these receptors is activated. NE signaling exerts its inhibitory effect on the proliferation of Th2 lymphocytes by binding to beta-adrenergic receptors. For example, the administration of the β2-adrenergic agonist clenbuterol during N. brasiliensis infection inhibits Th2 effector functions, leading to reduced recruitment of eosinophils and goblet cell hyperplasia [14].

Cytokines play a fundamental role as mediators of metabolic, hormonal, immunological, and hematological responses. The main pro-inflammatory cytokines involved in innate immunity are Tumor Necrosis Factor alpha and beta and Interleukins (ILs) 1-α, 1-β, IL-6, and IL-12, whose main cellular sources include macrophages, endothelial cells, dendritic cells, and T lymphocytes. Their biological effects include the activation of inflammation and coagulation, fever, catabolism, synthesis of acute phase proteins, and differentiation of CD4+ Th1 and Th17 T lymphocytes. On the other hand, among the anti-inflammatories, IL-10 stands out, synthesized by macrophages and regulatory T cells, with actions related to inhibiting the synthesis of ILs 1 and 12 and Tumor Necrosis Factor by macrophages and dendritic cells by negative feedback. There is therapeutic potential in blocking the cellular expression of these cytokines. However, there are many questions about this immunomodulation, and it is becoming increasingly necessary to elucidate the challenges that cytokines have left, not only for experimentation but also for clinical practice [15,16].

The protective innate immune responses during trauma produce a series of immediate responses to eliminate damaged tissues, followed by the activation of repair mechanisms, with the ultimate aim of restoring cells and tissues to their pre-lesion state. Pathogen-associated molecular patterns (PAMPs) of infectious agents (bacteria, viruses, and fungi) or the release of damage-associated molecular patterns (DAMPs) such as ATP, HMGB-1, histones, and mitochondrial DNA can be detected by fluid-phase inflammatory pathways that contain proteins or lipids and participate in the so-called “first line of defense”. In particular, the serine protease system, which participates in the kinin, coagulation, and complement cascades, can detect DAMPs and PAMPs, be rapidly activated after trauma, and be reinforced in acidic (e.g., hypoxic) microenvironments. Directly or via such activated systems, DAMPs and PAMPs can transmit their signals to leukocytes via pattern recognition receptors (PRRs) such as TLRs, NLRs, RAGE, purinergic receptors, or complement receptors. Cell translation and its interaction with the mechanisms described above result in broadly balanced pro-inflammatory and anti-inflammatory protective effects mediated by targeted chemotaxis and cytokine release that contribute to tissue repair and healing, such as the reprogramming of macrophages from the pro-inflammatory M1 phenotype to the anti-inflammatory M2 phenotype [17].

The impact of analgesic and surgical methods on the tissue levels of two representative cytokines, TNF and IL-10, which span the pro- and anti-inflammatory spectra, was demonstrated in an experimental study in which laparoscopic surgery for colorectal cancer surgery was linked to lower values of IL-10, TNF, and mast cells in the mucosa of the tissue surrounding the tumor, regardless of the method of perioperative analgesia. The number of IL-10-producing Th2 lymphocytes was found to be higher in patients undergoing open surgery compared to those undergoing laparoscopic surgery. Additionally, the number of TNF-producing cells was found to be higher in the open surgery group that received patient-controlled intravenous analgesia compared to the group that received epidural analgesia. The combination of laparoscopic surgery with epidural analgesia has been demonstrated to result in a reduction in the expression of TNF and IL-10, as well as mast cells. This approach may, therefore, represent a promising avenue for achieving anti-inflammatory effects in cancer-related surgical inflammation [18].

#### 3.1.2. Neuroendocrine and Humoral Response

**Figure 1:**
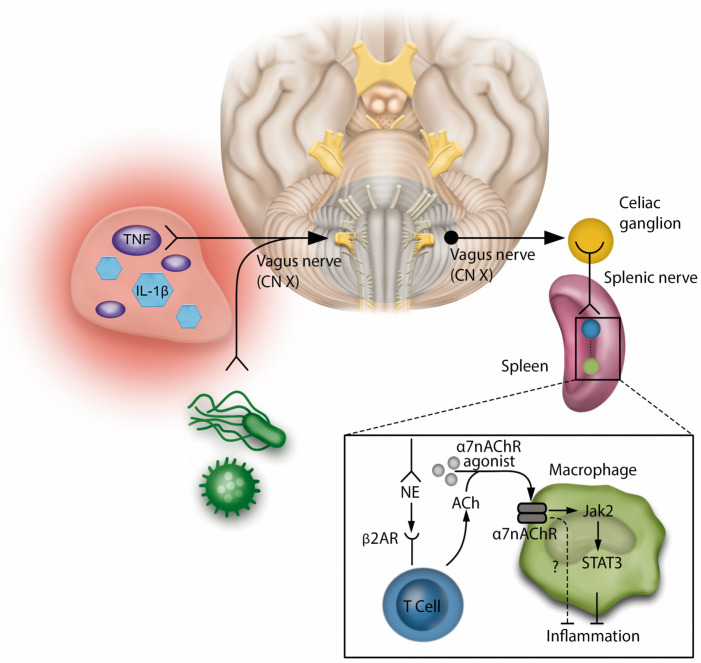
Neuroinflammatory reflexThe neuroinflammatory reflex involves interactions between sensory and motor pathways. Sensory afferents of the vagus nerve detect products of inflammation and infection. This information is relayed to the brainstem, processed by the central nervous system, and results in motor signals from the vagus nerve to the celiac ganglion, stimulating the splenic nerve. The splenic nerve ends near a specialized T Cell in the spleen that produces acetylcholine. Functioning similarly to an interneuron, the norepinephrine released by the splenic nerve activates the B2 adrenergic receptors on the T Cell, leading to the release of acetylcholine. This triggers the a7 nicotinic acetylcholine receptor on splenic macrophages, controlling Tumor Necrosis Factor production and promoting immunomodulation and anti-inflammation, Regulation of cytokine production by a7nAChR in immune cells involves signaling through Janus kinase 2 and signal transducer and activator of transcription 3. Pharmacological agonists of a7nAChR that stimulate the inflammatory reflex are being explored as anti-inflammatory treatments. NC stands for cranial nerve; IL-1β stands for interleukin-1β.

The neuroinflammatory reflex is activated by the sensory afferents of the vagus nerve, which are stimulated by the products of inflammatory and infectious stimuli. This information is transmitted to the brainstem, where it is integrated by the central nervous system. The reflex is completed by the vagus motor signals being sent to the celiac ganglion and splenic nerve. The latter ends near a specialized acetylcholine-producing T cell (ACh) in the spleen. The NE released by the splenic nerve activates the β2 adrenergic receptors (β2ARs) on the T cell, which in turn releases ACh. ACh activates the α7 nicotinic acetylcholine receptor (α7nAChR) in splenic macrophages and negatively regulates their production of Tumor Necrosis Factor (TNF). This mechanism appears to be mediated by the activation of Janus Kinase (JAK) 2 and transcriptional activator (STAT) 3 cell signaling, resulting in an anti-inflammatory effect (Figure 1). In this context, a series of experimental studies have demonstrated the therapeutic potential of modulating the neuroinflammatory pathway. Pharmacological agonists of α7nAChR, which activate the inflammatory reflex, are being developed as potential anti-inflammatory therapies, with promising results in experimental models for sepsis and surgical injury [2,3,19].

**Figure 2:**
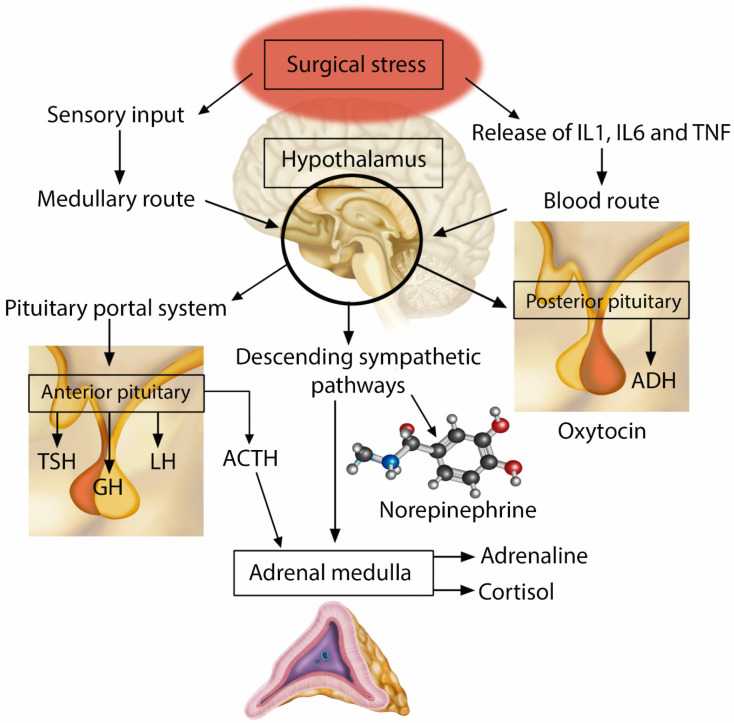
Neuroendocrine Response to Injury.

The parvocellular division of the hypothalamic paraventricular nucleus is the primary location of corticotrophin-releasing hormone (CRH) neurons and constitutes a key site for adaptive responses to stress. Sympathetic afferent stimuli arrive in the noradrenergic neurons of the brainstem located in the medulla oblongata (nucleus of the solitary tract—NTS), resulting in the activation and central regulation of the hypothalamic–pituitary–adrenal axis (Figure 2) [20]. CRH and arginine vasopressin are synthesized and released into the anterior pituitary gland by the pituitary portal vessels, which then promotes the synthesis of adrenocorticotropic hormone (ACTH). After release, ACTH binds to type 2 melanocortin receptors in the fasciculated zone of the adrenal cortex, triggering the synthesis and release of the primary stress hormones, glucocorticoids (cortisol). A neurological sympathetic response is initiated as an evolutionary form of survival, through a complex interaction between components of the central nervous system and peripheral systems, including the endocrine, immune, and cardiovascular systems [6].

Cortisol exerts its effects through its intranuclear, G-protein-coupled receptors, which are located in most organs, including the brain itself as part of negative feedback circuits. These receptors modulate and express numerous genes involved in mitochondrial metabolism, immune function, inflammation, growth, cognition, reproduction, and lung development. In the endothelium, for instance, the action of these receptors directly modulates endothelial physiology by regulating the expression of adhesion molecules (VCAM-1, ICAM-1, and E-selectin) and the production of pro-inflammatory cytokines and chemokines. These include cytokines (IL-6, IL-17F, and IL-8), vasodilators (nitric oxide), and vasoconstrictors (angiotensin II or endothelin-1), which are involved in maintaining endothelial morphology and reactivity [6].

The activation of the sympathetic nervous system (SNS) results in the stimulation of glucagon and inhibition of insulin release. Secretion of the anabolic hormone insulin is reduced by the effect of the SNS on pancreatic alpha-2 adrenergic receptors, which subsequently leads to a decrease in insulin sensitivity in peripheral cells. The hormonal changes that ensue result in hyperglycemia and the release of fatty acids with unopposed catabolism of muscle tissue [21].

The activation of humoral systems, including the coagulation and complement systems, occurs early after injury. The severity of the trauma and its outcome appear to be associated with elevated levels of anaphylatoxins C3a, C4a, and C5a. Acidic and hypoxic environments are known to activate the main complement factors, thus generating C3 and C5 activation products that can modulate intracellular mechanisms of lymphocytes. At lower concentrations, the metabolic effects of activated complement modulate CD4+ T cells in terms of glutamine utilization and increased T cell oxidative capacity. However, a higher concentration of complement results in cell death due to ATP depletion. Therefore, trauma alters the cellular responses of neutrophils, monocytes, and B cells, in addition to direct modulation of complement regulatory proteins in T cells and robust activation of C3a, C4a, and C5a [22].

Consequently, controlling the neuroendocrine and humoral response represents a pivotal strategy for regulating postoperative outcomes following trauma. Metabolic and hydroelectrolytic alterations resulting from the adrenergic response on the effector endocrine tissue can precipitate deleterious events in a susceptible organism. Therefore, multimodal anesthesia, which incorporates strategic drugs with specific mechanisms of action and regional blocks, is of paramount importance when this objective is pursued [23].

### 3.2. Modulation of the Inflammatory Response to Surgical Trauma—Therapeutic Interventions and Protective Strategies

The adrenergic tone on the immune system determines endocrine–metabolic changes and demonstrates the intercommunication between the neural, glandular effector, and immune systems. Therefore, there is a need to change the perspective of surgical injury treatment, which has previously been treated as pain, to a neuroinflammatory perspective. The anti-inflammatory effects of certain drugs may promote benefits in controlling SIRS and imply a favorable outcome in terms of the immediate postoperative period and early hospital discharge [24]. Research indicates that patients subjected to deep general anesthesia may exhibit reduced IL-6 levels 24 h post-surgery. However, this reduction does not correlate with positive outcomes on peripheral T lymphocyte and natural killer cell subsets in laparoscopic surgery for colorectal cancer, irrespective of using a BIS of 55 or 35 [25].

#### Anesthetic Drugs

− Dexmedetomidine

Dexmedetomidine exerts its effects on the locus coeruleus and spinal cord, inhibiting the pre-synaptic release of NE. This results in sedation, analgesia, and a central sympatholytic effect. It is an excellent perioperative option as it produces beneficial effects in reducing the response to inflammatory and hemodynamic stress generated by surgical trauma, with minimal side effects that could be generated when other drugs are used (benzodiazepines and opioids) [2,3].

A single-blinded, randomized, controlled clinical trial investigated the effect of dexmedetomidine on T helper 1 (Th1) and T helper 2 (Th2) cytokines and their ratio during and after laparoscopic cholecystectomy in ASA I and II patients. Th1 cells produce IFN-γ and favor cell-mediated immune responses. Th2 cells produce IL-4 and/or IL-10 and favor humoral immunity. Macrophages express alpha-2 adrenoreceptors on their surfaces, stimulating the production of IL-12, a potent inducer of Th1 cells. This study showed that intraoperative administration of dexmedetomidine attenuated the immune response induced by surgical stress by increasing the IFN-γ/IL-4 ratio (Th1/Th2 ratio) [26]. Another study also showed an increase in the Th17/Treg lymphocyte ratio, i.e., a decrease in the production of IL-4 and IL-10, which are Treg cell cytokines, and a shift in the balance towards Th17. Dexmedetomidine treatment also prevented an increase in CRP levels after surgery, indicating that the use of α-2 agonists can modulate the immune response during and after laparoscopy [26].

Studies have demonstrated the efficacy of dexmedetomidine in controlling postoperative pain. It is associated with improved quality of postoperative recovery and reduced opioid consumption in the immediate postoperative period. These factors make dexmedetomidine an attractive agent for enhanced recovery in surgery (ERAS) protocols and for patients with acute and chronic pain. Furthermore, it can be used in conjunction with other pharmaceutical agents to prolong the duration and the quality of analgesia in peripheral, regional, and neuroaxis blocks [27,28,29].

− ketamine

Ketamine exerts its effects at various levels of inflammation, including the recruitment of inflammatory cells, cytokine production, and immunomodulation. Its analgesic properties are well characterized and mainly attributed to non-competitive inhibition of glutamate and aspartate receptors. However, the pharmacological targets of ketamine are not limited to NMDARs. It has been demonstrated that ketamine interacts with other receptors and ion channels, including dopamine, serotonin, sigma, opioid, and cholinergic receptors, as well as cyclic nucleotide-gated (HCN) channels activated by hyperpolarization [30]. Patients with treatment-resistant depression and coexisting pain exhibited a higher antidepressant response and remission rate than patients without associated pain. In the aforementioned group, the levels of TNF-α and IL-6 on day 13 and IFN-γ, IL-10, IL-1β, IL-4, IL-6, TNF-α, and other chemokines on day 26 were found to be lower than at the commencement of the study. In the group without pain, the levels of TNF-α on day 13 and 26 were observed to be lower than at the commencement of the study. The observed changes were primarily in IL-6, which were associated with improved pain intensity and depressive symptoms following ketamine treatment [31]. In addition, studies on video laparoscopic surgery have demonstrated that the intravenous administration of ketamine, administered preemptively and perioperatively, resulted in a reduction in pain scores immediately following surgery. Its analgesic effect is not sustained in the late postoperative period, and there is a positive impact on postoperative nausea and vomiting [32,33].

− Opioids

There is a reciprocal interaction between the immune system and endogenous/exogenous opioids. These substances are among the most potent analgesics in the treatment of severe pain, despite the risk of induced hyperalgesia, respiratory depression, nausea, and suppression of the immune response, which has the potential to increase vulnerability to infections. Exogenous opioids, including morphine, fentanyl, and sufentanil have been demonstrated to impair the function of macrophages, natural killer cells, and T cells, as well as to weaken the intestinal barrier in vitro. Epidemiological studies have also indicated that high doses and the initiation of opioid therapy for non-malignant pain are correlated with an increased risk of infectious diseases such as pneumonia [34].

It is postulated that there are centrally mediated mechanisms, as evidenced by the observation that opioids that cross the blood–brain barrier (BBB) exert more immunomodulatory effects than those that do not. While the effects of opioids are largely attributed to decreased outflow from the central sympathetic nervous system, opioids can also cause direct sympathetic nerve activation, which can suppress the proliferation and function of some immune cell populations and lymphoid tissues. Opioids attenuate the circadian rhythm of ACTH and cortisol, leading to consistent increases in circulating levels of these hormones, which may be sufficient to produce immunosuppression. Thus, long-term exposure to short-acting opioids (MOP-r agonists such as heroin or fentanyl) results in pathophysiological changes in neuroimmune and neuroinflammatory functions, affected, in part, by peripheral mechanisms (e.g., cytokines) and neuroendocrine systems such as the hypothalamic–pituitary–adrenal (HPA) stress axis [35]. However, this study has indicated that opioid-sparing anesthesia has a lower incidence of postoperative complications than opioid-based anesthetic techniques in video-assisted surgery [36].

− Peripheral Regional and Neuraxial Blocks

The capacity of neuronal blockade to modify the response to surgical trauma has been extensively investigated in recent years. For instance, the blockade of the Quadratus Lumborum Anterior has been demonstrated to attenuate the production of the cytokine Interleukin-6 and decrease the release of cortisol, accompanied by a significant attenuation of the surgical repercussions on pulmonary function and a reduction in postoperative pain scores and opioid consumption in laparoscopic cholecystectomy [37]. The use of perioperative epidural analgesia is potentially associated with fewer postoperative surgical complications. A retrospective, observational cohort study of patients who underwent pancreaticoduodenectomy showed reduction in post-operative surgical complications, such as the use of analgesics, antiemetics, antipyretics, blood transfusions and parenteral nutrition [38]. Thus, the proposed benefits of regional techniques include an earlier return of gut function, a reduced incidence of pulmonary dysfunction, and a lower inflammatory response to surgery.

### 3.3. Ischemic and Pharmacologic Preconditioning

The literature describes protective methods that can be employed to reduce unfavorable clinical outcomes related to excessive inflammatory response and organ damage. These interventions prepare cells for upcoming damage and prevent the activation of inflammatory cells/release of inflammatory mediators. Remote ischemic preconditioning (RIP) confers protection against renal ischemia–reperfusion injury in patients undergoing laparoscopic partial nephrectomy. This protection was observed in both the early and late phases, with the latter being more prominent. Serum neutrophil gelatinase-associated lipocalin (NGAL) and serum cystatin C (CysC) were lower after limb ischemic preconditioning [39]. This organ protection measure in patients undergoing laparoscopic colorectal cancer resection decreased the incidence of postoperative gastrointestinal dysfunction and lowered the IL-6, TNF-α, and I-FABP levels, demonstrating protective effect on patients’ postoperative gastrointestinal function [40].

A cohort of men undergoing robot-assisted laparoscopic prostatectomy for localized prostate cancer associated with pelvic inflammation exhibited elevated levels of the IL-6, STAT3, and IFN genes, indicating a potential role in STAT3 gene signaling. Autologous primary prostate cells or cancer cell lines were inhibited by silencing STAT3 and IL-6 signaling using fludarabine, a STAT3 inhibitor, and tocilizumab, an IL-6 inhibitor. The study indicated that the development of inhibitors of STAT-IL6 pathway signaling could help to mitigate the effects of inflammation-induced carcinogenesis [41]. In kidney transplantation, ischemia and reperfusion injury is a significant concern, with the potential to negatively impact the outcome of the graft and the patient. Commonly used volatile anesthetics, such as sevoflurane and isoflurane, have been shown to interfere with many of the pathophysiological processes involved in the reperfusion injury cascade. This cascade is characterized by dysfunction of the mitochondrial respiratory chain and uncontrolled formation of reactive oxygen species during reperfusion, leading to the opening of mitochondrial permeability transition pores and the release of danger-associated molecular patterns (DAMPs) into the intra- and extracellular space. Inhalants prevent the opening of mitochondrial pores; have protective effects on the glycocalyx; confer upregulation of Hypoxia-Inducible Factors, facilitating cellular adaptation to low oxygen conditions; and generate a positive effect on circulating immune cells [42].

It is crucial to highlight that the selection of an anesthetic regimen plays a pivotal role in reducing the size of ischemic heart lesions through pharmacological preconditioning. A study demonstrated that propofol effectively negated the preconditioning effect of milrinone and levosimendan, whereas sevoflurane exhibited no such effect. However, under the influence of dexmedetomidine, the outcomes were inconclusive [43].

### 3.4. Endothelial Glycocalyx Protection

**Figure 3:**
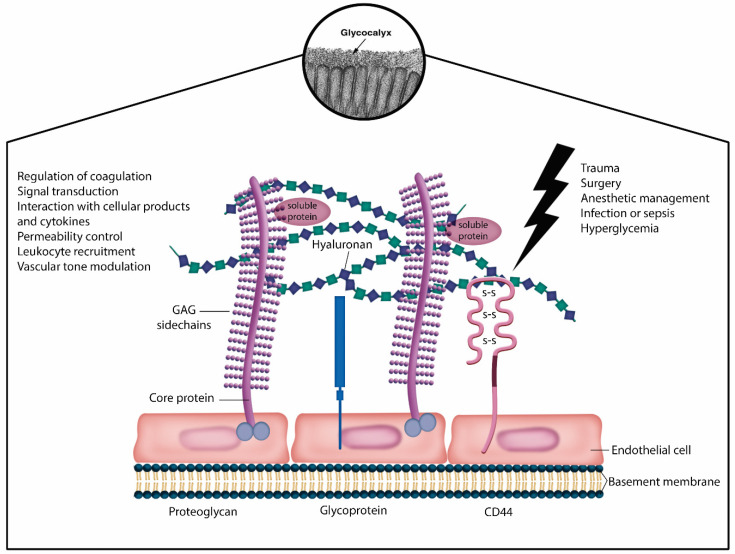
Endothelial glycocalyx structureSchematic representation of the glycocalyx enveloping endothelial cells shows that the endothelial glycocalyx (EG) primarily consists ofproteoglycans (syndecans and glypicans) with glycosaminoglycan side chains and glycoproteins (selectins and integrins). Components suchdas hyaluronan, plasma proteins, and soluble proteoglycans constitute the endothelial surface layer. The EG plays a crucial role in regulatingvascular function and homeostasis. Its integrity loss is associated with various inflammatory conditions, including trauma, infectious diseases, sepsis, diabetes mellitus, and hyperhydration.

The maintenance of endothelial glycocalyx integrity has the potential to modulate systemic inflammation. The glycocalyx functions as a sensor of mechanotransduction, physical protection barrier, signal transduction, and a source of interaction between cellular products and pro-inflammatory mediators (Figure 3). It has been linked to several crucial physiological processes within microcirculation, including blood coagulation, immunity, antioxidation, and interactions with serum proteins and sodium. In sepsis management, the approach to glycocalyx protection includes colloid substitution, preferably albumin and fresh frozen plasma; catecholamine restriction; restrictive fluid therapy; corticosteroids; anticoagulants; glycemic control; and vitamin C [44,45].

This study was conducted to investigate the association between immediate postoperative serum levels of syndecan-1, a representative marker of endothelial degradation, and severe postoperative morbidity and mortality in patients undergoing robot-assisted esophagectomy. A high level of syndecan-1 in the immediate postoperative period (≥48 ng/mL) was found to be associated with an increased risk of morbidity and mortality within 30 days of surgery. Patients with syndecan-1 levels ≥48 ng/mL exhibited a higher incidence of unexpected returns to the operating room and anastomotic leaks, as well as longer hospital and ICU stays than patients with syndecan-1 levels <48 ng/mL [46]. In addition to the modality and magnitude of the surgical trauma, acutely injured patients can develop secondary injury, mainly caused by continuous tissue trauma during surgical preparation, inflammatory reaction, hypovolemia due to hemorrhage, and other causes. While some interventions have been identified as having the potential to protect the glycocalyx, such as plasma transfusion, human serum albumin, hydrocortisone, and sevoflurane, there is currently no specific treatment for the protection and recovery of this barrier in clinical medicine that can be used during the perioperative period. However, the literature highlights strategies to minimize its impairment in the surgical environment. These include performing damage control surgery to remove potential sources of sepsis, minimizing surgical time, restoring and maintaining hemodynamic stability, and avoiding water overload [47]. The current experimental and clinical evidence indicates a potential for clinical interventions that can modulate endothelial dysfunction, such as the administration of its structural components, including sphingosine-1-phosphate, hyaluronan, and sulodeoxide, as well as the combination of medium-long chain heparan sulfate and dermatan sulfate. However, there is a need for properly designed and robust clinical trials to support routine use in acutely compromised patients [48] (Table 1).

## 4. Biomarkers

The included studies investigated potential blood biomarkers to determine their correlation with the extent of surgical injury, anesthetic intervention, administered drugs, and the inflammatory state. This review indicates that therapeutic approaches could affect immunomodulatory processes either indirectly, by regulating the activity of immune cells, or directly, by mitigating the effects of stress. The extent of this influence varies depending on the clinical or surgical situation under consideration. The main biomarkers used are listed in Table 2.

There is a trend to use some biomarkers that are more sensitive to tissue injury as they alter the balance between pro- and anti-inflammatory responses to a greater extent. Each marker presents an individual behavior with fluctuations over time.

## 5. Conclusions

Several sustainable therapeutic approaches, when combined, can significantly reduce inflammation in response to infection or surgery/trauma, potentially improving patient outcomes and decreasing healthcare-related costs. Protective approaches in medical treatments, such as dexmedetomidine, ketamine, opioid-sparing techniques, interventions for ischemic and pharmacologic preconditioning, strategies to maintain the integrity of the endothelial glycocalyx, and peripheral and neuroaxis blocks, represent therapeutic avenues in the capable hands of anesthesiologists and critical care physicians. Biofluid biomarkers have proven to be clinically valuable in both diagnosis and prognosis. Their systematic categorization serves as a roadmap for investigating their behavior following interventions in cases of injury, making them an indispensable tool. The effects of immunomodulating inflammation approaches are multifaceted. While perioperative immunosuppression can heighten the risk of tumor metastasis and infection, the anti-inflammatory effects of various drugs and strategies may still contribute positively to conditions linked with systemic inflammation. In the future, research on advanced gene expression analysis may eventually pinpoint population groups with increased inflammatory vulnerability in the perioperative context, leading to improved clinical outcomes.

## Figures and Tables

**Table 1:** Overview of anesthetic drugs and therapeutic strategies and their impact on surgical stress response.

Drugs and Therapeutic Strategies	Outcomes and Clinical Implications
Dexmedetomidine	Decreases the inflammatory response and hemodynamic stress generated by surgical trauma, with minimal side effects [2,3]; Increases the IFN-γ/IL-4 ratio (Th1/Th2 ratio) [26]; Increases the Th17/Treg lymphocyte ratio, i.e., a decrease in the production of IL-4 and IL-10; also prevented an increase in CRP levels [27]; Postoperative pain control; decreases opioid consumption in the immediate postoperative period; and prolongs the duration and the quality of analgesia in peripheral regional and neuroaxis blocks [27,28,29].
Ketamine	Interacts with NMDA receptors; ion channels, including dopamine, serotonin, sigma, opioid, and cholinergic receptors; and cyclic nucleotide-gated (HCN) channels activated by hyperpolarization [31]; Lowers levels of TNF-α, IL-6, IFN-γ, IL-10, IL-1β, and IL-4 in patients exhibiting depressive symptoms and associated pain [31]; Reduction in pain scores following surgery; positive impact on postoperative nausea and vomiting [32,33].
Opioids	Immunosuppressive effects impairing monocyte and neutrophil function, NK-cell-mediated; potential correlation exists between this condition and the recurrence of tumors after cancer surgery [34]; Inhibits the secretion of CRH, ACTH, and cortisol [35]; Opioid-sparing anesthesia has a lower incidence of postoperative complications [36].
Peripheral Regional and Neuraxial Blocks	Quadratus Lumborum Anterior blockade attenuates Interleukin-6 and decreases the release of cortisol [37]; Attenuation of surgical repercussions on pulmonary function; decreases postoperative pain scores and opioid consumption [37]; Epidural analgesia reduces the use of analgesics, antiemetics, antipyretics, blood transfusions, and parenteral nutrition [38].
Ischemic and Pharmacologic Preconditioning	Protection against renal ischemia–reperfusion injury; lower levels of serum neutrophil gelatinase-associated lipocalin (NGAL) and serum cystatin C [39]; Decreases the incidence of postoperative gastrointestinal dysfunction; lowers the IL-6, TNF-α, and I-FABP levels [40]; Inhibitors of STAT-IL6 pathway signaling mitigate the effects of inflammation-induced carcinogenesis [41]; Sevoflurane and isoflurane interfere with the pathophysiological processes involved in the reperfusion injury cascade [42], reducing the size of ischemic heart lesions with milrinone and levosimendan [43].
Endothelial Glycocalyx Protection	Colloid substitution, preferably with albumin and fresh frozen plasma; catecholamine restriction; restrictive fluid therapy; corticosteroids; anticoagulants; glycemic control; and vitamin C can offer glycocalyx protection [44,45].

**Table 2:** Biomarkers and main characteristics.

Inflammatory Markers	Characteristics Of Its Systemic Effects
TNF-α	Earliest and most potent pro-inflammatory cytokine after tissue trauma; Peak effect time of 1 h and half-life of 20 min.; Activates the cytokine release cascade, including IL-6; Activates coagulation.
IL1-β	Pro-inflammatory cytokine; Synergism with TNF-α; Half-life of 6 min; Fever response to trauma.
IL-2	Induces production of immunoglobulins and T lymphocytes; Half-life of 10 min.
IL-6	Main cytokine with pro- and anti-inflammatory effects; Detectable after 60 min with peak effect between 4 and 6 h; Stimulates production of CRP; IL-6 levels correspond to the extent of tissue trauma; Anti-inflammatory effect of counter-regulation of TNF-α and IL1-β activity.
IL-10	Anti-inflammatory cytokine; Modulates TNF Alfa activity; Peak effect in 3 h.
IFN-γ	Pro-inflammatory cytokine that induces IL-2, IL-12, and IL-18 production; Activation of macrophages; Detectable within 6 h and persists elevated for 8 days.
CRP	Anti- and pro-inflammatory activities; Related to the extent of surgical trauma; Acute-phase protein whose hepatic production is induced by IL-6; Detectable after 4–6 h, with a peak at 48 h.
Cortisol	Activated by IL-6 secretion and is considered an acute-phase hormonal reactant; Associated with the degree of surgical trauma; Peaks approximately 4–6 h after incision.
TP/ TTP/ TAPT	Times of coagulation shortened, reflecting the hypercoagulable state.
Adrenaline/Norepinephrine	Adrenaline reflects the activity of the adrenal medulla; Noradrenaline reflects the activity of the sympathetic nervous system; Half-life of 2–3 min under physiological conditions; Induces catabolism, hyperglycemia, and proteolysis; Peaks approximately 15 min from the onset of trauma.

## Data Availability

Not applicable.

## References

[1] Silva G.N., Brandão V.G., Perez M.V., Lewandrowski K.-U., Fiorelli R.K.A. (2023). Effects of Dexmedetomidine on Immunomodulation and Pain Control in Videolaparoscopic Cholecystectomies: A Randomized, Two-Arm, Double-Blinded, Placebo-Controlled Trial. J. Pers. Med..

[2] Silva G.N., Brandão V.G., Fiorelli R., Perez M.V., Mello C.R., Negrini D., Levandrowski K.-U., Martinelli R.B., dos Reis T.P.D.A. (2023). Outcomes of dexmedetomidine as adjuvant drug in patients undergoing videolaparoscopic cholecystectomy: A randomized and prospective clinical trial. Int. J. Immunopathol. Pharmacol..

[3] Brandão V.G.A., Silva G.N., Perez M.V., Lewandrowski K.-U., Fiorelli R.K.A. (2023). Effect of Quadratus Lumborum Block on Pain and Stress Response after Video Laparoscopic Surgeries: A Randomized Clinical Trial. J. Pers. Med..

[4] Geng Z., Bi H., Zhang D., Xiao C., Song H., Feng Y., Cao X., Li X. (2021). The impact of multimodal analgesia based enhanced recovery protocol on quality of recovery after laparoscopic gynecological surgery: A randomized controlled trial. BMC Anesthesiol..

[5] Dobson G.P. (2020). Trauma of major surgery: A global problem that is not going away. Int. J. Surg..

[6] Burford N.G., Webster N.A., Cruz-Topete D. (2017). Hypothalamic-pituitary-adrenal axis modulation of glucocorticoids in the cardiovascular system. Int. J. Mol. Sci..

[7] Margraf A., Ludwig N., Zarbock A., Rossaint J. (2020). Systemic inflammatory response syndrome after surgery: Mechanisms and protection. Anesth. Analg..

[8] Silva G.N., Brandão V.G., Perez M.V., Sobrinho S.L., Villardi J.G.d.C.C., Sacramento P.M.D., Ribeiro L.C.P., Fiorelli R.K.A. (2024). Immunotherapeutic Properties of Dexmedetomidine on Pain Management and Cardiovascular Function in Videolaparoscopic Cholecystectomies: A randomized, two-arm, double-blinded, placebo-controlled trial. Surg. Innov..

[9] Watt D.G., Horgan P.G., McMillan D.C. (2015). Routine clinical markers of the magnitude of the systemic inflammatory response after elective operation: A systematic review. Surgery.

[10] Floros T., Philippou A., Bardakostas D., Mantas D., Koutsilieris M. (2016). The growth endocrine axis and inflammatory responses after laparoscopic cholecystectomy. Hormones.

[11] Page A.J., Ejaz A., Spolverato G., Zavadsky T., Grant M.C., Galante D.J., Wick E.C., Weiss M., Makary M.A., Wu C.L. (2015). Enhanced recovery after surgery protocols for open hepatectomy--physiology, immunomodulation, and implementation. J. Gastrointest. Surg..

[12] Curry N., Brohi K. (2020). Surgery in Traumatic Injury and Perioperative Considerations. Semin. Thromb. Hemost..

[13] Hotamisligil G.S., Davis R.J. (2016). Cell Signaling and Stress Responses. Cold Spring Harb. Perspect. Biol..

[14] Thurairajah K., Briggs G.D., Balogh Z.J. (2018). The source of cell-free mitochondrial DNA in trauma and potential therapeutic strategies. Eur. J. Trauma Emerg. Surg..

[15] Cortez V.S., Robinette M.L., Colonna M. (2015). Innate lymphoid cells: New insights into function and development. Curr. Opin. Immunol..

[16] Eberl G., Colonna M., Di Santo J.P., McKenzie A.N.J. (2015). Innate lymphoid cells. Innate lymphoid cells: A new paradigm in immunology. Science.

[17] Huber-Lang M., Lambris J.D., Ward P.A. (2018). Innate immune responses to trauma. Nat. Immunol..

[18] Faisal M., Schäfer C.N., Myrelid P., Winberg M.E., Söderholm J.D., Keita V., Eintrei C. (2021). Effects of analgesic and surgical modality on immune response in colorectal cancer surgery. Surg. Oncol..

[19] Steinberg B.E., Sundman E., Terrando N., Eriksson L.I., Olofsson P.S. (2016). Neural Control of Inflammation: Implications for Perioperative and Critical Care. Anesthesiology.

[20] Barman S.M. (2020). 2019 Ludwig Lecture: Rhythms in sympathetic nerve activity are a key to understanding neural control of the cardiovascular system. Am. J. Physiol. Integr. Comp. Physiol..

[21] Cusack B., Buggy D.J. (2020). Anaesthesia, analgesia, and the surgical stress response. BJA Educ..

[22] Chakraborty S., Karasu E., Huber-Lang M. (2018). Complement After Trauma: Suturing Innate and Adaptive Immunity. Front. Immunol..

[23] Cruz F.F., Rocco P.R.M., Pelosi P. (2017). Anti-inflammatory properties of anesthetic agents. Crit. Care.

[24] Kaye A.D., Chernobylsky D.J., Thakur P., Siddaiah H., Kaye R.J., Eng L.K., Harbell M.W., Lajaunie J., Cornett E.M. (2020). Dexmedetomidine in Enhanced Recovery After Surgery (ERAS) Protocols for Postoperative Pain. Curr. Pain Headache Rep..

[25] Li H., Li J., Hao C., Luan H., Zhang X., Zhao Z. (2023). Effects of anesthetic depth on perioperative T lymphocyte subsets in patients undergoing laparoscopic colorectal cancer surgery: A prospective, parallel-controlled randomized trial. BMC Anesthesiol..

[26] Lee J.-M., Han H.-J., Choi W.-K., Yoo S., Baek S., Lee J. (2018). Immunomodulatory effects of intraoperative dexmedetomidine on T helper 1, T helper 2, T helper 17 and regulatory T cells cytokine levels and their balance: A prospective, randomised, double-blind, dose-response clinical study. BMC Anesthesiol..

[27] Vorobeichik L., Brull R., Abdallah F.W. (2017). Evidence basis for using perineural dexmedetomidine to enhance the quality of brachial plexus nerve blocks: A systematic review and meta-analysis of randomized controlled trials. Br. J. Anaesth..

[28] Yazdi B., Modir H., Piri M., Almasi-Hashiani A. (2021). An investigation of the effects of dexmedetomidine and fentanyl as an adjuvant to ropivacaine on pain scores and hemodynamic changes following laparoscopic cholecystectomy. Med. Gas Res..

[29] Sen I.M., Prashanth K., Bhatia N., Goel N., Kaman L. (2021). Paravertebral block using levobupivacaine or dexmedetomidine-levobupivacaine for analgesia after cholecystectomy: A randomized double-blind trial. Braz. J. Anesthesiol..

[30] Zanos P., Moaddel R., Morris P.J., Riggs L.M., Highland J.N., Georgiou P., Pereira E.F.R., Albuquerque E.X., Thomas C.J., Zarate C.A. (2018). Ketamine and Ketamine Metabolite Pharmacology: Insights into Therapeutic Mechanisms. Pharmacol. Rev..

[31] Zhou Y., Wang C., Lan X., Li H., Chao Z., Ning Y. (2021). Plasma inflammatory cytokines and treatment-resistant depression with comorbid pain: Improvement by ketamine. J. Neuroinflamm..

[32] Hung K.-C., Wu S.-C., Chang P.-C., Chen I.-W., Hsing C.-H., Lin C.-M., Chen J.-Y., Chu C.-C., Sun C.-K. (2021). Impact of Intraoperative Ketamine on Postoperative Analgesic Requirement Following Bariatric Surgery: A Meta-analysis of Randomized Controlled Trials. Obes. Surg..

[33] Jain S., Nazir N., Mustafi S. (2022). Preemptive low-dose intravenous ketamine in the management of acute and chronic postoperative pain following laparoscopic cholecystectomy: A prospective randomized control study. Med. Gas Res..

[34] Plein L.M., Rittner H.L. (2018). Opioids and the immune system—Friend or foe. Br. J. Pharmacol..

[35] Butelman E.R., Goldstein R.Z., Nwaneshiudu C.A., Girdhar K., Roussos P., Russo S.J., Alia-Klein N. (2023). Neuroimmune Mechanisms of Opioid Use Disorder and Recovery: Translatability to Human Studies, and Future Research Directions. Neuroscience.

[36] Jiang Y.-Y., Li Z.-P., Yao M., Zhou Q.-H. (2022). Standard opioid-containing versus opioid-sparing anesthesia on early postoperative recovery after video-assisted thoracic surgery: A propensity-weighted analysis. Front. Surg..

[37] Brandão V.G.A., Silva G.N., Fiorelli R.K.A., Perez M.V. (2023). Outcome of Ultrasound Guided Anterior Quadratus Lumborum Block after Video Laparoscopic Cholecystectomies: A Prospective Randomized Clinical Trial. Surg. Innov..

[38] Negrini D., Ihsan M., Freitas K., Pollazzon C., Graaf J., Andre J., Linhares T., Brandao V., Silva G., Fiorelli R. (2022). The clinical impact of the perioperative epidural anesthesia on surgical outcomes after pancreaticoduodenectomy: A retrospective cohort study. Surg. Open Sci..

[39] Hou Y.-Y., Li Y., He S.-F., Song J., Yu D.-X., Wong G.T., Zhang Y. (2017). Effects of differential-phase remote ischemic preconditioning intervention in laparoscopic partial nephrectomy: A single blinded, randomized controlled trial in a parallel group design. J. Clin. Anesth..

[40] Yi M., Wu Y., Li M., Zhang T., Chen Y. (2023). Effect of remote ischemic preconditioning on postoperative gastrointestinal function in patients undergoing laparoscopic colorectal cancer resection. Int. J. Color. Dis..

[41] Chakravarty D., Ratnani P., Huang L., Dovey Z., Sobotka S., Berryhill R., Merisaari H., Al Shaarani M., Rai R., Jambor I. (2022). Association between Incidental Pelvic Inflammation and Aggressive Prostate Cancer. Cancers.

[42] Nieuwenhuijs-Moeke G.J., Bosch D.J., Leuvenink H.G. (2021). Molecular Aspects of Volatile Anesthetic-Induced Organ Protection and Its Potential in Kidney Transplantation. Int. J. Mol. Sci..

[43] Bunte S., Lill T., Falk M., Stroethoff M., Raupach A., Mathes A., Heinen A., Hollmann M.W., Huhn R. (2019). Impact of Anesthetics on Cardioprotection Induced by Pharmacological Preconditioning. J. Clin. Med..

[44] Astapenko D., Benes J., Pouska J., Lehmann C., Islam S., Cerny V. (2019). Endothelial glycocalyx in acute care surgery—What anaesthesiologists need to know for clinical practice. BMC Anesthesiol..

[45] Iba T., Maier C.L., Helms J., Ferrer R., Thachil J., Levy J.H. (2024). Managing sepsis and septic shock in an endothelial glycocalyx-friendly way: From the viewpoint of surviving sepsis campaign guidelines. Ann. Intensive Care.

[46] Kim H.J., Choi Y.S., Park B.J., Shin H.J., Jeon S.Y., Kim D.J., Kim S.Y. (2023). Immediate Postoperative High Syndecan-1 is Associated with Short-Term Morbidity and Mortality After Robot-Assisted Esophagectomy: A Prospective Observational Study. Ann. Surg. Oncol..

[47] Kravitz M.S., Kattouf N., Stewart I.J., Ginde A.A., Schmidt E.P., Shapiro N.I. (2024). Plasma for prevention and treatment of glycocalyx degradation in trauma and sepsis. Critical Care.

[48] Mathis S., Putzer G., Schneeberger S., Martini J. (2021). The Endothelial Glycocalyx and Organ Preservation-From Physiology to Possible Clinical Implications for Solid Organ Transplantation. Int. J. Mol. Sci..

